# Heat Shock Protein 40 (HSP40) in Pacific White Shrimp (*Litopenaeus vannamei*): Molecular Cloning, Tissue Distribution and Ontogeny, Response to Temperature, Acidity/Alkalinity and Salinity Stresses, and Potential Role in Ovarian Development

**DOI:** 10.3389/fphys.2018.01784

**Published:** 2018-12-12

**Authors:** Ting Chen, Tiehao Lin, Hongmei Li, Ting Lu, Jiaxi Li, Wen Huang, Hongyan Sun, Xiao Jiang, Jiquan Zhang, Aifen Yan, Chaoqun Hu, Peng Luo, Chunhua Ren

**Affiliations:** ^1^CAS Key Laboratory of Tropical Marine Bio-resources and Ecology, Guangdong Provincial Key Laboratory of Applied Marine Biology, South China Sea Institute of Oceanology, Chinese Academy of Sciences, Guangzhou, China; ^2^South China Sea Bio-Resource Exploitation and Utilization Collaborative Innovation Center, Guangzhou, China; ^3^Guangdong Institute for Drug Control, Guangzhou, China; ^4^College of Marine Sciences, South China Agricultural University, Guangzhou, China; ^5^Foshan University, Foshan, China; ^6^College of Life Sciences, Hebei University, Baoding, China

**Keywords:** heat shock protein, HSP40, tissue distribution and ontogeny, environmental stress, ovarian development, crustacean

## Abstract

Heat shock proteins (HSPs), a family of conserved proteins that are produced by cells in response to stresses, are known as molecular chaperones with a range of housekeeping and cellular protective functions. The 40 kD heat shock protein (HSP40) is a co-chaperone for HSP70 in the regulation of ATP hydrolysis. Unlike its well-documented cofactor HSP70, little is currently known regarding the biological functions of HSP40 in crustacean species such as penaeid shrimp. In the present study, the cDNA encoding HSP40 (*Lv*-HSP40) was identified from the Pacific white shrimp *Litopenaeus vannamei*, a highly significant commercial culture species. The structural organization indicates that Lv-HSP40 belongs to the type-I HSP40s. The muscle, gill, and hepatopancreas are the main sites of *Lv-HSP40* transcript expression. Within these tissues, *Lv-HSP40* mRNA were predominantly exhibited in the myocytes, epithelial cells and hepatopancreatic cells, respectively. Under acute thermal stress in the culture environment, *Lv-HSP40* transcript levels are significantly induced in these three tissues, while low pH stress only upregulates *Lv-HSP40* mRNA in the hepatopancreas and gill. During ontogenesis, *Lv-HSP40* transcript levels are high at early embryonic stages and drop sharply at late embryonic and early larval stages. The ovary is another major organ of *Lv-HSP40* mRNA expression in female shrimp, and *Lv-HSP40* transcripts were mainly presented in the follicle cells but only weekly detected in the oocytes. Ovarian *Lv-HSP40* mRNA levels increase continuously during gonadal development. Silencing of the *Lv-HSP40* gene by RNA interference may effectively delay ovarian maturation after unilateral eyestalk ablation. The roles of Lv-HSP40 in ovarian development are speculated to be independent of its cofactor HSP70, and the vitellogenesis factor vitellogenin (*Vg*) and vitellogenin receptor (*VgR*). Our study, as a whole, provides new insights into the roles of HSP40 in multiple physiological processes in *L. vannamei*: (1) HSP40 is a responding factor during stressful conditions; and (2) HSP40 participates in embryonic and ovarian development.

## Introduction

Heat shock proteins (HSPs), also known as heat stress proteins, are a family of proteins that are produced by cells in response to various stressful conditions (Zhu et al., 1996; Johnston et al., 2018). HSPs were first identified in fruit flies that were exposed to a severely heat-shocked environment (Ritossa, 1962) and were subsequently demonstrated to be ubiquitously and evolutionarily conserved molecular chaperones that are present in all living organisms (Srivastava, 2002). Mostly according to their molecular weights, HSPs can be categorized into different families, including the HSP100, HSP90, HSP70, HSP60, HSP40 families and several small HSP families (Feder and Hofmann, 1999). In addition to thermal stress, different kinds of environmental and pathological stresses, such as toxins, oxidative conditions, hypoxia, glucose deprivation, water deprivation, osmotic pressure, infection and inflammation, can also trigger high levels of intracellular HSP production (Santoro, 2000; Srivastava, 2002; Wang et al., 2017; Xu et al., 2017). Moreover, HSPs may perform a multitude of housekeeping functions that are essential for cell survival (Srivastava, 2002). Under normal conditions, HSPs participate in cellular processes such as protein folding and transport, cell cycle regulation and apoptosis (Mallouk et al., 1999; Johnston et al., 2018), as well as in physiological processes such as embryonic development, gonadal development and spermatogenesis (Allen et al., 1996; Binder, 2014; Chan et al., 2014).

The 40 kD heat shock protein (HSP40, also called DnaJ) is an HSP subfamily, each member in which contains the J domain, a 70-amino acid (a.a.) domain, with similarity to the N-terminus of *Escherichia coli* DnaJ (Georgopoulos et al., 1980; Zylicz et al., 1985). The conserved J domain is necessary for HSP40 binding to HSP70 (also called DnaK) (Kelley, 1998), another molecular chaperone that couples the cycles of ATP binding, hydrolysis and ADP release. As the co-chaperone of HSP70, HSP40 is capable of tightly regulating ATP hydrolysis, which is necessary for many normal housekeeping and stress-related functions (Fan et al., 2003). In addition to the N-terminal J domain, the other two major domains in HSP40 are the central cysteine-rich (C/R) domain, which acts in a zinc-dependent fashion (Martinez-Yamout et al., 2000), and the C-terminal (CT) domain, which is for chaperoning and dimerization (Lu and Cyr, 1998). Functionally, HSP40s have been reported to participate in DNA binding, protein degradation, intracellular signal transduction, exocytosis, endocytosis, viral infection, apoptosis and sensing heat shock, nitric oxide, and ischemic stresses (Cheetham and Caplan, 1998).

The Pacific white shrimp *Litopenaeus vannamei* is the most widely cultured and productive crustacean species, nearly 71% of the global economic penaeid shrimp production (Alfaro-Montoya, 2010). However, the development of the *L. vannamei* aquaculture industry in recent years has been seriously affected by various harsh culture environmental parameters, such as water temperature (Huang et al., 2017), acidity/alkalinity (Lemonnier et al., 2004; Cai et al., 2017), salinity (McGraw and Scarpa, 2004; Chen et al., 2016b), nutrition (Romano and Zeng, 2012) and disease exposure (Chen et al., 2016a; Tian et al., 2018a,b). The reproductive performance of shrimp is currently regarded as another major drawback in *L. vannamei* aquaculture (Alfaro-Montoya, 2010; Chen et al., 2014; Luo et al., 2015). To solve the existing and emerging problems in shrimp culture, the biological and physiological functions of HSPs have been investigated in a variety of economic shrimp species. In *Penaeus monodon*, *HSP10*, *HSP21*, *HSP60*, *HSP70*, and *HSP90* transcripts were inducible expressed under either thermal, pH challenge, salinity, and heavy metal exposure or bacterial challenge conditions (Jiang et al., 2009; Rungrassamee et al., 2010; Shi et al., 2016). In *Macrobrachium rosenbergii*, *HSP60*, *HSP70*, and *HSP90* mRNA patterns were stimulated selectively after bacterial or viral infection (Chaurasia et al., 2016). In *Metapenaeus ensis*, HSC70 (the constitutive form of HSP70) was found to be a negative regulator of vitellogenesis (Chan et al., 2014). In *L. vannamei*, *HSP10*, *HSP27*, *HSP30*, *HSP60*, *HSP70* and *HSP90* mRNA were consistently or specifically expressed in response to thermal or pH stress, heavy metal exposure (Qian et al., 2012), and bacterial or viral infection (Junprung et al., 2017; Yuan et al., 2017); HSP70 promoted post-larval tolerance to heat, ammonia and metals (Sung et al., 2018), and HSC70 reduced protein aggregation and mortality after viral infection (Yuan et al., 2017).

Unlike well-characterized HSPs such as HSP70, little knowledge is available regarding the cofactor HSP40 in crustaceans, despite the fact that the responses of HSP40 to environmental stress and pathogenic infection have been described in some non-crustacean aquatic species, such as the halibut *Paralichthys olivaceus* (Dong et al., 2006), clam *Venerupis philippinarum* (Li et al., 2011) and oyster *Pinctada martensii* (Li J. et al., 2016). To investigate the roles of HSP40 in various physiological processes of *L. vannamei*, we first identified the full-length cDNA of *HSP40* from the hepatopancreas of *L. vannamei*. Tissue distribution and ontogeny of *HSP40* mRNA was analyzed by quantitative real-time PCR. Changes of *HSP40* mRNA expression in the hepatopancreas, muscle and gill were detected after acute temperature, pH and salinity challenges. Moreover, the potential role of HSP40 in ovarian development was investigated by the RNA interference (RNAi) approach.

## Materials and Methods

### Animals

The adults, embryos and larvae of Pacific white shrimp (*L. vannamei*) were collected from the Dongfang shrimp culture center, Zhanjiang, Guangdong, China, and they were maintained in artificial seawater [30 parts per thousand (ppt) and pH 8.2] at 28°C under a 12-h dark:12-h light photoperiod. The shrimp were fed every morning, and feces and other detritus in each tank were siphoned out after feeding. Sexually immature shrimp with body weights of 7.2 ± 0.9 g and body lengths of 7.3 ± 0.6 cm were used for the molecular cloning and stress challenge experiments. Shrimp were anesthetized on ice and killed by decapitation. All animal experiments were conducted in accordance with the guidelines and approval of the Ethics Committees of the South China Sea Institute of Oceanology, Chinese Academy of Sciences.

### Molecular Cloning and Bioinformatics Analysis of Lv-HSP40

Total RNA from the shrimp hepatopancreas was extracted using TRIzol reagent (Invitrogen), digested with DNase I (Invitrogen) and reversely transcribed into first-strand cDNA with PrimeScript^TM^ II Kit (TaKaRa). Based on a unigene of 1075 bp (Supplementary Data 1) that was obtained from an Illumina transcriptome of *L. vannamei* constructed by our lab previously (Huang et al., 2017) and shares high sequence homology with HSP40 in other species, gene-specific primers were designed to amplify a partial sequence of *L. vannamei HSP40* (*Lv-HSP40*). The corresponding full-length sequence was obtained by 3′- and 5′-rapid amplification of cDNA ends (RACE).

Structural domains were predicted with the SMART and ScanProsite programs. Three-dimensional (3-D) model was generated by using the SWISS-MODEL server. Phylogenetic analysis for different crustacean HSPs was performed by the neighbor-joining method with 1000 bootstrap replicates using MEGA 6.0.

### Tissue Distribution and Ontogeny of *Lv-HSP40* mRNA

The tissue distribution of *L. vannamei HSP40* mRNA was quantitatively detected in six individuals. The selected tissues included eyestalk, brain, thoracic nerve, abdominal nerve, gill, heart, hepatopancreas, hemolymph, muscle, leg, stomach and intestine from sexually immature shrimp, as well as ovary and testis, respectively, from female and male shrimp during gonadal development. The embryos and larvae were sampled in nine developmental stages according to their morphologies as observed using an optical microscope, including zygote, blastula, gastrula, limb bud embryo, larva in membrane, nauplius, zoea, mysis, and post-larvae. Embryos and larvae were collected when 80% of the population had reached the objective stage, and the morphology for each stage was determined as described previously (Wei et al., 2014). The tissue, embryonic and larval samples were frozen immediately in liquid nitrogen, then stored at -80°C prior to further analysis of mRNA expression.

### *In situ* Hybridization of *Lv-HSP40* mRNA Expressed Cells

The cellular localization of *HSP40* mRNA were performed in selected tissues with high transcript levels by *in situ* hybridization (*IS*H). Briefly, samples from the muscle, gill, hepatopancreas and ovary were removed quickly and fixed overnight in 4% paraformaldehyde in PBS. Samples were dehydrated in an ethanol series, immersed in xylene, embedded in paraffin, cut into 5 μm sections and mounted onto slides. A digoxin (DIG)-labeled DNA probe of 585 bp (Supplementary Data 2) against *Lv-HSP40* mRNA was synthesized with the DIG DNA Labeling Kit (Roche). The sections were deparaffinized, prehybridized and hybridized with the DIG-labeled DNA probe (0.4 ng/ml) at 42°C overnight as described previously (Chen et al., 2008). After that, the sections were incubated with horseradish peroxidase (HRP)-conjugated anti-DIG antibody (Roche), and the *IS*H signal were developed by a diaminobenzidine (DAB, MXB Biotechnologies) reaction and observed with a Leica DM-IRB light microscopy (Leica).

### Temperature, Acidity/Alkalinity and Salinity Challenge

The changes in the mRNA expression of *L. vannamei HSP40* and its cofactor *HSP70* were measured in shrimp under temperature, acidity/alkalinity and salinity stresses. After acclimation in artificial seawater (30 ppt and pH 8.2) at 28°C for 2 weeks, fifty-five shrimp were randomly transferred into nine independent 20-L tanks (6 individuals per tank). The challenge conditions for shrimp included low temperature (13°C) and high temperature (37°C) [as validated by (Huang et al., 2017)], low pH (pH 6.8) and high pH (pH 8.9) [as validated by (Cai et al., 2017)], and low salinity (10 ppt) and high salinity (45 ppt) [as validated by (Chen et al., 2016b)]. Six shrimp from each group were killed at 6 h after challenge. The hepatopancreas, muscle and gill samples were collected for further analysis, as described above.

### Ovarian Development of Female Shrimp

Previtellogenic female shrimp, with body weights of 48.4 ± 1.6 g, body lengths of 18.3 ± 0.3 cm and a gonadosomatic index (GSI) <0.3%, were applied for artificially induced maturation with unilateral eyestalk ablation and nutrition strength (Chen et al., 2014). Ovarian development was defined into four stages (stage I-IV) based on the classification of predominant oocytes as described previously (Tinikul et al., 2011; Chen H.-Y. et al., 2018). Briefly, the anterior end of ovarian explants with the number 2–9 lobes (Bae et al., 2017) were fixed overnight in 4% paraformaldehyde. The samples were dehydrated through an ethanol series, immersed in xylene and embedded in paraffin. The sections were cut into 5 μm sections, mounted onto slides and stained with hematoxylin and eosin (H/E). In stage I, the ovaries are mainly comprised of oogonia and previtellogenic oocytes; in stage II, the ovaries majorly compose previtellogenic oocytes and endogenous vitellogenetic oocytes; in stage III, the ovaries process a large number of oocytes developing from endogenous vitellogenetic oocytes to exogenous vitellogenetic oocytes; in stage IV, exogenous vitellogenetic oocytes and mature oocytes predominate in the ovaries. In this study, the GSIs for shrimp in stage I, II, III and IV were 0.3–0.9%, 0.9–2.5%, 2.5–5.0% and >5.0%, respectively. The hepatopancreatic and ovarian samples from six individuals of each stage were collected for further analysis of *HSP40* and *HSP70* mRNA expression, as described above.

### Gene Silencing of Lv-HSP40 mRNA

The effects of HSP40 on ovarian development were investigated using an RNAi approach. Small interfering RNA (siRNA) targeting sites were identified using the Ambion siRNA target finder program, and the selected sequences were subjected to global BLAST against non-redundant nucleotide sequences, as described previously (Cai et al., 2017). Three sets of siRNA (HSP40-42, HSP40-474, and HSP40-825, Supplementary Data 3) specific against *Lv-HSP40* and the non-targeting nematode siRNA (NC-siRNA) were synthesized by Sangon Biotech Company. To determine the knockdown efficiencies, hepatopancreatic and ovarian *Lv-HSP40* transcripts were measured by real-time PCR at 12 and 24 h after siRNA injection. Given that injection of siRNA HSP40-474 with a dosage of 300 ng/g body weight (bwt) showed the highest mRNA knockdown efficiency, it was selected for the subsequent *Lv-HSP40* gene silencing experiments.

Previtellogenic female shrimp with body sizes as described above were used for RNAi experiments. The injection of siRNA was conducted as described before (Cai et al., 2017). Briefly, one-hundred fifty shrimp were transferred randomly into three independent 1-m^3^ tanks (fifty individuals per tank). Each shrimp was injected with 100 μL of siRNA HSP40-474 (300 ng/g bwt) in PBS, and either injection of PBS alone or NC-siRNA (300 ng/g bwt) in PBS were used as control samples. The shrimp in each group were sampled at 1, 2, 3, 4, and 5 days after siRNA injection. The GSIs were weighted and calculated after sampling, and the ovarian developmental stages were confirmed by the observation of paraffin sections with H/E staining. In parallel experiments, the mRNA levels of the cofactor *HSP70* and the vitellogenesis essential genes *vitellogenin* (*Vg*) in the hepatopancreas and ovaries, and vitellogenin receptor (*VgR*) in the ovaries from the shrimp at 24 h after siRNA injection were measured by real-time PCR.

### Measurement of mRNA Expression for Target Genes

The transcript levels of target genes (*HSP40*, *HSP70*, *Vg*, and *VgR*) were measured in multiple tissues, embryonic, larval developmental stages, and ovarian developmental stages, as well as under different challenge/treatment conditions, by quantitative real-time PCR. Briefly, 2 μg total RNA was extracted using TRIzol reagent, digested with DNase I and reversely transcribed into first-strand cDNA with PrimeScript^TM^ II Kit. The cDNA samples obtained were then subjected to quantitative PCR performed on a Roche Light-Cycler 480 system (Corbett Research) by using SYBR Premix Ex Taq^TM^ II (Takara) according to the manufacturer’s protocol (Supplementary Data 4). In this study, β*-actin* was used as an internal control. Serially diluted plasmid DNAs containing the *HSP40*, *HSP70*, *Vg, VgR* (Supplementary Data 5) and β*-actin* sequences were used as the standards for real-time PCR. After the PCR reactions, the identities of PCR products were routinely confirmed by melting curve analysis. The raw data were expressed in terms of fmol target transcript detected per tube, and the data were routinely normalized as a ratio of β*-actin* mRNA detected in the same sample.

### Data Transformation and Statistical Analysis

For mRNA expression, the raw data were simply transformed as a percentage of the mean values in the control group. The statistical analysis were performed with SPSS (IBM Software). For mRNA and GSI measurement, the data expressed as the mean ± S.E. (standard error) were analyzed by using Student’s *t*-test or one-way ANOVA followed by Fisher’s least significant difference (LSD) test. For staging of ovarian development, the data were examined by using Ridit analysis. Differences were considered significant at *P* < 0.05.

## Results

### Molecular Cloning and Bioinformatic Analysis of *Lv-HSP40* cDNA

**FIGURE 1:**
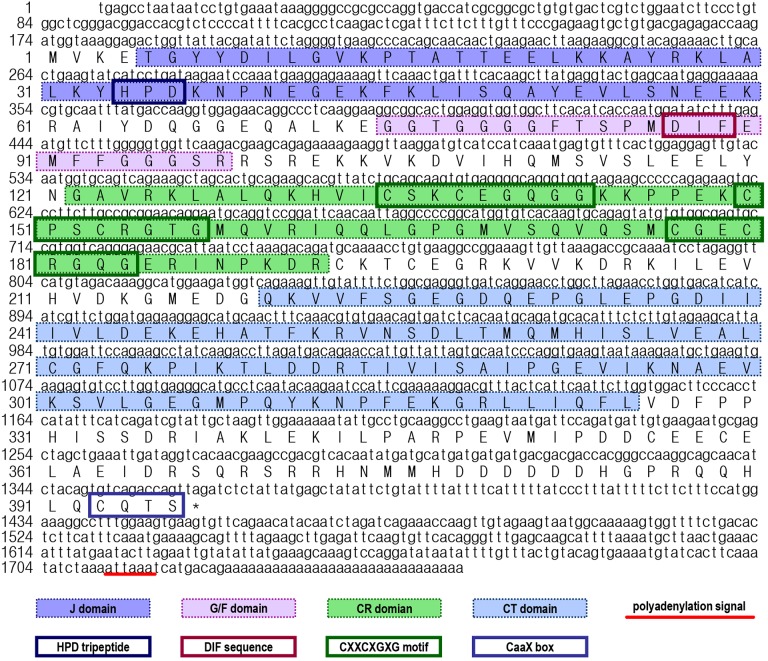
Nucleotide and deduced amino acid sequences of *Litopenaeus vannamei* HSP40 cDNA. The a.a. sequences deduced from the ORF are presented along with the corresponding cDNA sequence. The polyadenylation signal in the 3′-UTR is underlined, and the stop codon is marked by an asterisk. In the a.a. sequence, the N-terminal J domain, glycine/phenylalanine-rich (G/F) domain, cysteine-rich (C/R) domain, C-terminal (CT) domain, HPD tripeptide, DIF sequence, CXXCXGXG motifs and CaaX box are marked with different symbols.

In this study, the full-length cDNA for a heat shock protein 40 (*Lv-HSP40*) was identified from the Pacific white shrimp. As shown in Figure 1, the *Lv-HSP40* cDNA (GenBank: MH932106) is 1756 bp in size, with a 173-bp 5′-untranslated region (UTR), a 391-bp 3′-UTR region and a 1191-bp open reading frame (ORF) coding for a 396-a.a. protein precursor (∼44.4 kDa). In the 3′-UTR, a polyadenylation signal (attaaa) is located 15-bp upstream of the poly-A tail.

**FIGURE 2:**
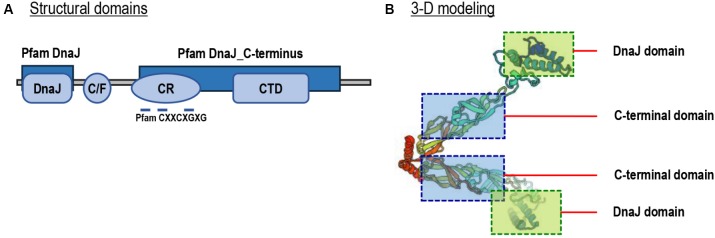
**(A)** Structural domains of Lv-HSP40 predicted by the SMART and ScanProsite programs. The J, G/F, C/R, and CT domains are boxed, and the CXXCXGXG motifs are indicated. **(B)** Three-dimensional (3-D) protein model for a Lv-HSP40 dimer by the SWISS-MODEL server. The J domains and CT domains are boxed and indicated.

**FIGURE 3:**
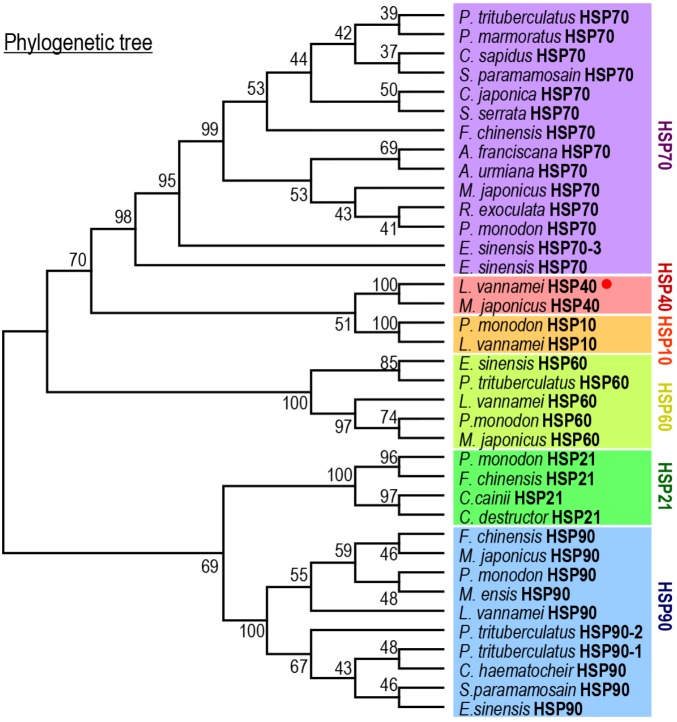
Phylogenetic analysis of HSPs among various crustacean species using the neighbor-joining method with a bootstrap value of 1000.

By prediction using the SMART and ScanProsite programs, a DnaJ domain, a glycine/phenylalanine-rich (G/F) domain, a Zn^2+^-binding CR domain and a CT domain were found in the deduced Lv-HSP40 protein sequence (Figures 1, 2A). The J domain is 56-a.a. in length and is where a highly conserved HPD tripeptide is located. Adjacent to the J-domain is a 24-a.a. G/F domain with a conserved DIF sequence. A 106-a.a. CR domain, composed of three repeats of the CXXCXGXG motif, is next to the G/F domain. In the C- terminus, a 106-a.a. CT domain and a CaaX motif are present. In addition, a 3-D model of the Lv-HSP40 dimer showed that the J domains were located in the exterior part, and the CT domains were located in the inner part (Figure 2B). Furthermore, a phylogenetic tree built with several HSPs in crustacean species showed that HSP70, HSP40, HSP10, HSP21, and HSP90 were grouped into respective branches, and our newly cloned Lv-HSP40 was clustered with the HSP40 from lobster *Metanephrops japonicas* (Figure 3).

### Expression Profiles of *Lv-HSP40* mRNA in Different Tissues and in Different Embryonic and Larval Stages

**FIGURE 4:**
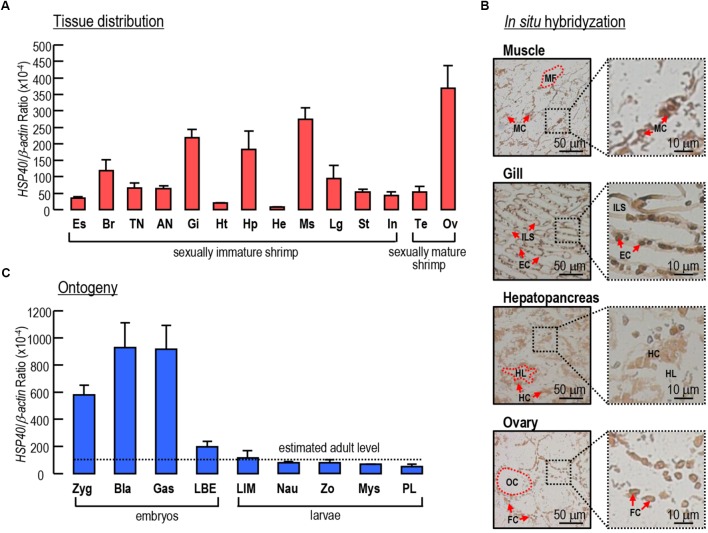
**(A)** Tissue distribution of *Lv-HSP40* mRNA. The selected tissues include eyestalk (Es), brain (Br), thoracic nerve (TN), abdominal nerve (AN), gill (Gi), heart (Ht), hepatopancreas (Hp), hemolymph (He), muscle (Ms), leg (Lg), stomach (St), intestine (In), testis (Te), and ovary (Ov). **(B)** *In situ* hybridization of *Lv-HSP40* mRNA expression cells in the muscle, gill, hepatopancreas and ovary. The cell types and tissue structures in *IS*H include myocytes (MC), myofibril (MS), epithelial cells (EC), inter lamellar space (ILS), hepatopancreatic cells (HC), tubule lumen (TL), oocytes (OC), and follicle cells (FC). The scale bars for the overall and partial pictures were 50 μm and 10 μm, respectively. **(C)** Ontogeny of *Lv-HSP40* mRNA. The selected embryonic and larval developmental stages include the zygote (Zyg), blastula (Bla), gastrula (Gas), limb bud embryo (LBE), larva in membrane (LIM), nauplius (Nau), zoea (Zo), mysis (Mys) and post-larval (PL) stages. For tissue distribution and ontogeny, the data presented are expressed as the mean ± S.E. (*n* = 6).

The transcript expression profiles of *Lv-HSP40* were detected in multiple tissues by quantitative PCR. As shown in Figure 4A, *Lv-HSP40* mRNA could be ubiquitously detected in all tissues we examined. The highest expression level of *Lv-HSP40* mRNA was observed in the ovaries of female shrimp, and relatively high expression levels were detected in the muscle, gill, and hepatopancreas. In addition, an *IS*H for *Lv-HSP40* mRNA expressed cells were performed in the tissues with high transcript levels (Figure 4B). In this case, *Lv-HSP40* mRNA were predominantly detected in the myocytes, epithelial cells and hepatopancreatic cells in the muscle, gill and hepatopancreas, respectively. In the ovary, the *Lv-HSP40* mRNA were mainly presented in the follicle cells with only weak signals in the oocytes.

During embryonic and larval development, the expression of *Lv-HSP40* mRNA showed high levels at the stages of zygote, blastula and gastrula, then reduced sharply at the stage of limb bud embryo and remained at relatively low levels at the stages of larva in membrane, nauplius, zoea, mysis and post-larvae (Figure 4C). During early embryonic development, the *Lv-HSP40* transcript levels were much higher than the estimated average level (mean value of *Lv-HSP40* mRNA among different tissues) in adults, while during late embryonic development and larval development, the *Lv-HSP40* transcript levels were similar to the estimated average level in adults (Figure 4C).

### Response of *Lv-HSP40* mRNA Expression Under Temperature, Acidity/Alkalinity and Salinity Stress

**FIGURE 5:**
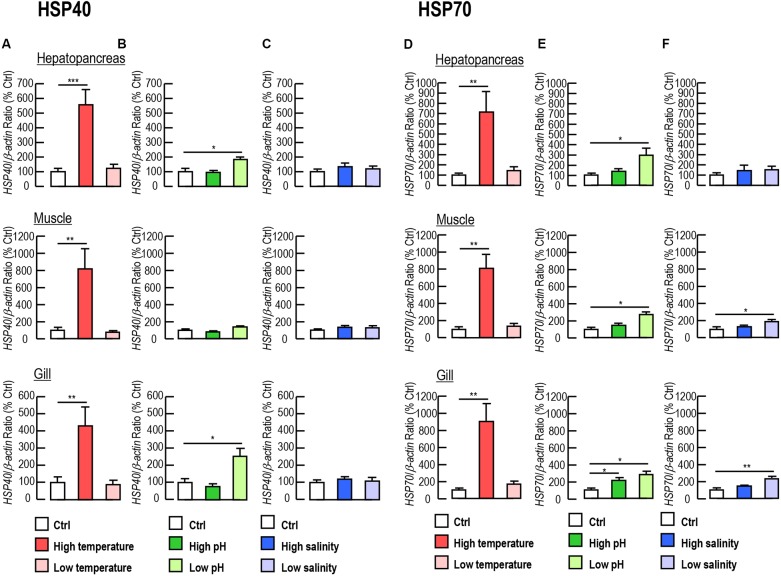
**(A–C)** *Lv-HSP40* mRNA expression in the hepatopancreas, muscle and gill of *L. vannamei* under challenges of high or low temperature **(A)**, high or low pH **(B)**, and high or low salinity **(C)**. **(D–F)** *Lv-HSP70* mRNA expression in the hepatopancreas, muscle and gill of *L. vannamei* under challenges of high or low temperature **(D)**, high or low pH **(E)**, and high or low salinity **(F)**. In these cases, the data presented are expressed as the mean ± S.E. (*n* = 6), and significant differences between the control and challenge groups were assessed using Student’s *t*-test (^∗^*P* < 0.05, ^∗∗^*P* < 0.01 and ^∗∗∗^*P* < 0.001).

Given that *Lv-HSP40* showed high transcript levels in the hepatopancreas, muscle and gill of shrimp, the responses of *Lv-HSP40* mRNA under temperature, acidity/alkalinity and salinity stresses were detected in these tissues. The expression levels of *Lv-HSP40* mRNA in the hepatopancreas, muscle and gill significantly increased when shrimp were transferred to a high temperature (37°C, Figure 5A) but showed no change when they were transferred to a low temperature (13°C, Figure 5A). Under low pH challenge (pH 6.8, Figure 5B), *Lv-HSP40* mRNA increased in the hepatopancreas and gill but remained stable in the muscle. In contrast, *Lv-HSP40* mRNA did not respond to high pH stress (pH 8.9, Figure 5B) in any tissues we tested. Additionally, *Lv-HSP40* mRNA levels in all tissues examined neither changed under high salinity (45 ppt, Figure 5C) nor low salinity stress (10 ppt, Figure 5C). In parallel experiments, mRNA levels of *Lv-HSP70* were upregulated in the hepatopancreas, muscle and gill under high temperature (Figure 5D) and low pH (Figure 5E) challenges, in the gill under high pH challenge (Figure 5E), and in the muscle and gill under low temperature challenge (Figure 5F). In contrast, *Lv-HSP70* mRNA expression was stable in other stress groups we tested (Figures 5D–F).

### Roles of Lv-HSP40 in Shrimp Ovarian Development

**FIGURE 6:**
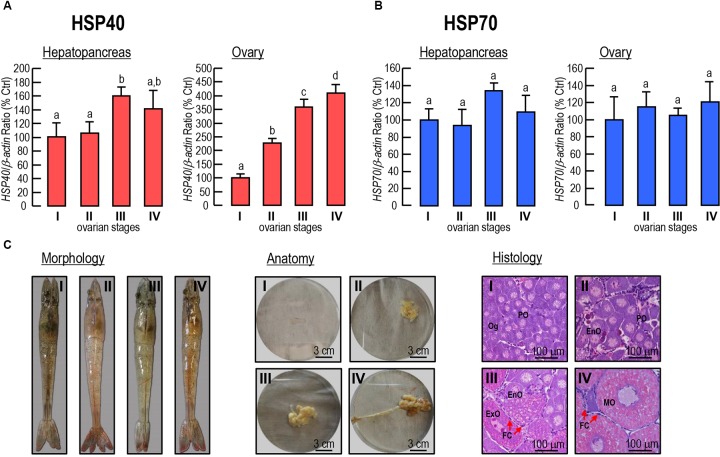
**(A,B)** Hepatopancreatic and ovarian *Lv-HSP40* **(A)** and *Lv-HSP70* **(B)** transcript levels in female shrimp in different ovarian developmental stages. For mRNA expression, the data presented are expressed as the mean ± S.E. (*n* = 6), and experimental groups denoted by the same letter represent a similar level (*P* > 0.05, ANOVA followed by Fisher’s LSD test). **(C)** Morphology, anatomy and histology of the stage I, II, III and IV ovaries in *L. vannamei*. The cells in histological sections include oogonia (Og), previtellogenic oocytes (PO), endogenous vitellogenetic oocytes (EnO), exogenous vitellogenetic oocytes (ExO), mature oocytes (MO) and follicle cells (FC). The scale bars for the anatomic and histological pictures were 3 cm and 100 μm, respectively.

The expression levels of *Lv-HSP40* mRNA were detected in the hepatopancreas and ovary from female shrimp in different ovarian developmental stages (stages I–IV). In the hepatopancreas, *Lv-HSP40* mRNA was stable at stages I and II, slightly increased in stage III and reduced to the normal level at stage IV (Figure 6A). In the ovary, *Lv-HSP40* mRNA increased acutely and continuously from stage I to stage IV (Figure 6A). In contrast, the *Lv-HSP70* (Figure 6B) transcript levels in the hepatopancreas and ovary remained stable throughout gonadal development in female shrimp. In this case, the morphology, anatomy and histology of *L. vannamei* ovaries for staging were shown in Figure 6C.

**FIGURE 7:**
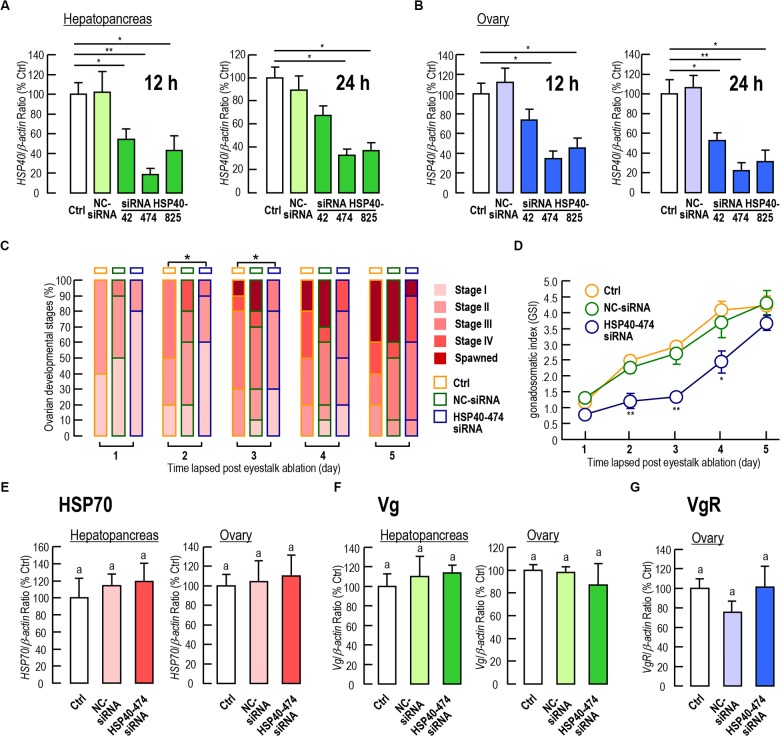
**(A,B)** Knockdown efficiencies of siRNA HSP40-42, HSP40-474, HSP40-825 on the *Lv-HSP40* mRNA expression in the hepatopancreas **(A)** and ovary **(B)** at 12 h and 24 h after injection. **(C)** Percentages of ovarian development stages (stage I, II, III, IV, and spawned) for female shrimp at 1, 2, 3, 4, and 5 days after the injection of PBS (Ctrl), NC-siRNA, or HSP40-474 siRNA. **(D)** Gonadosomatic index (GSI) for female shrimp at 1, 2, 3, 4 and 5 days after the injection of PBS (Ctrl), NC-siRNA or HSP40-474 siRNA. **(E–G)** Hepatopancreatic and ovarian *Lv-HSP70* **(E)** and *Lv-Vg* **(F)**, and ovarian *Lv-VgR* **(G)** transcript levels of female shrimp at 24 h after the injection of PBS (Ctrl), NC-siRNA or HSP40-474 siRNA. For staging of ovarian development, the data presented were examined using Ridit analysis (^∗^*P* < 0.05). For GSI measurements, the data presented are expressed as the mean ± S.E. (*n* = 10), and significant differences between the PBS and siRNA injection groups were assessed using Student’s *t*-test (^∗^*P* < 0.05 and ^∗∗^*P* < 0.01). For mRNA expression, the data presented are expressed as the mean ± S.E. (*n* = 6), and experimental groups denoted by the same letter represent a similar level (*P* > 0.05, ANOVA followed by Fisher’s LSD test).

Among the three siRNA we tested, siRNA HSP40-474 showed the highest knockdown efficiency on *Lv-HSP40* mRNA levels in both the hepatopancreas (Figure 7A) and ovary (Figure 7B), and no significant mortality was observed with siRNA injection. After injection of siRNA HSP40-474, the ovarian developments of female shrimp were delayed when compared to that in the PBS and NC-siRNA injection groups, based on the observation of the ovarian stages (Figure 7C) with significant differences (*P* < 0.05) by Ridit analysis were shown at 2 and 3 day after injection. Spawning in the *HSP40* siRNA injection group was also delayed but not totally abolished (Figure 7C). Similarly, the increasing of GSI induced by eyestalk ablation in the *HSP40* siRNA injection group was also slower than that in the PBS and NC-siRNA injection groups (Figure 7D). In parallel experiments, the injection of HSP40 siRNA for 24 h did not alter the *HSP70* (Figure 7E), *Vg* (Figure 7F) and *VgR* (Figure 7G) mRNA levels in either the hepatopancreas or ovary.

## Discussion

In this study, the full-length cDNA of a novel HSP40 has been identified from Pacific white shrimp, *L. vannamei*. The newly obtained Lv-HSP40 contains complete J domain, G/F domain, CR domain and CT domain (Figure 2A). There are three types of HSP40s categorized according to their domain organization. Type-I HSP40s possess J domain, G/F domain, CR domain and CT domain; type-II HSP40s lack the zinc binding CR domain; and type-III HSP40s retain only the J domain (Dong et al., 2006). Therefore, the structural features indicate that Lv-HSP40 belongs to the type-I HSP40s. In addition, the deduced 3-D structure of the Lv-HSP40 dimer (Figure 2B) shows that the CT domains from two monomers are joined together for dimerization (Lu and Cyr, 1998), while the J domains are located externally for binding to HSP70 (Kelley, 1998).

The tissue distribution showed that *Lv-HSP40* mRNA was widely expressed in all tissues we investigated, with high transcript levels observed in the muscle, gill and hepatopancreas in sexually immature shrimp and, especially, in the ovary in sexually mature female shrimp (Figure 4A). By *IS*H, the positive signals of *Lv-HSP40* mRNA were exhibited in the main cell types of muscle, gill and hepatopancreas, respectively (Figure 4B), suggesting the extensive functions of Lv-HSP40 in multiple tissues. In contrast, *Lv-HSP40* transcripts were majorly detected in the follicle cells but not oocytes, implying that a role of Lv-HSP40 in the ovary may be related to the supply of nutrients and energy. The tissue expression profiles of *HSP40* transcripts are diverse in different animal species. In the silkmoth *Bombyx mori*, the highest HSP40 protein level was detected in the hemocytes, followed by the body and head (Li J. et al., 2016). In the grasshopper *Oxya chinensis*, high levels of *HSP40* mRNA were observed in the Malpighian tubule, followed by the brain and fat body (Zhang et al., 2015). Interestingly, the *HSP* mRNA tissue distribution in the marine teleost fish *P. olivaceus* was similar to that in *L. vannamei*, in which the liver, muscle, gill and ovary are the major tissues with *HSP* mRNA expression (Dong et al., 2006).

The ontogeny of *Lv-HSP40* mRNA was further detected in our study. As shown in Figure 4C, *Lv-HSP40* mRNA levels were high at early embryonic stages, such as the zygote, blastula and gastrula stages; during the transition from late embryos (limb bud embryo stage) to early larva (larva in membrane stage), the expression of *Lv-HSP40* decreased rapidly; during late larval stages, the *Lv-HSP40* transcripts remained at relative low levels that are similar to the estimated average level in adults. Based on the mRNA expression pattern, it is speculated that, in early embryonic developmental stages in which yolk is used as the nutritional source, more molecular chaperones are required for energy generation by hydrolyzing ATP into ADP (Zhu et al., 1996; Mallouk et al., 1999). Meanwhile, a large number of proteins produced in embryonic stages also need HSPs to serve as molecular chaperones to assist in folding or unfolding and in assembly or disassembly (Zhu et al., 2010). Similar findings for the functional roles of HSP40 in embryonic and juvenile development have been previously demonstrated in the mouse (Zhu et al., 2010), grasshopper *O. chinensis* (Zhang et al., 2015) and crustacean rotifer *Brachionus calyciflorus* (Yang et al., 2014).

HSPs are well-known for their quick response to environmental stresses (Lewis et al., 2016; Sung et al., 2018). However, the respective roles of HSP40 in response to a particular environmental stress have not been illustrated in *L. vannamei* to date, even though the expression profiles of other HSPs in *L. vannamei*, such as *HSP60*, *HSP70* and *HSP90*, were reported under acute thermal stress, pH challenge and heavy metal exposure (Qian et al., 2012). In both wild marine and aquaculture pond environments, *L. vannamei* are constantly exposed to various environmental stresses that can result in biochemical, physiological, and histological alterations. Our current study showed that the expression levels of *Lv-HSP40* mRNA rose in response to low pH challenge in the hepatopancreas and gill, in addition to showing a response to high temperature stress (Figures 5A–C), indicating that HSP40 is an effector gene in *L. vannamei* under multiple aquatic stresses. The involvement of HSP genes in the heat-shock response has been described in several crustacean species, such as HSP70 and HSP90 in *P. monodon* (Li et al., 2009); HSP70, HSC70 and HSP90 in *Fenneropenaeus chinensis* (Luan et al., 2010); HSP70 and HSC70 in *M. rosenbergii* (Liu et al., 2004); and HSP60, HSP70 and HSP90 in *L. vannamei* (Qian et al., 2012). The involvements of HSP60, HSP70 and HSP90 in *L. vannamei* have further been demonstrated by their acidity and alkalinity responses (Qian et al., 2012). In addition, the salinity response has been reported in *P. monodon* with HSP21 (Shekhar et al., 2013) and in *Portunus trituberculatus* with HSP60 (Xu and Qin, 2012). In our parallel experiments, the responses of Lv-HSP70 under various stresses were tested (Figures 5D–F), and the results were similar but not identical to those of previous reports (Qian et al., 2012), which may be owing to the shrimp sizes, treatment durations or experimental conditions that were different between these two studies. In oyster *P. martensii*, transcript expression of *HSP40* showed similar kinetics as that of *HSP70* in response to high temperature and low salinity stress (Li J. et al., 2016). However, this phenomenon was not observed in *L. vannamei* in our present study.

The tissue distribution showed that *Lv-HSP40* mRNA was highly expressed in ovary (Figure 4A), implying a possible role of HSP40 during the gonadal development of female shrimp. In *M. ensis*, previous studies showed that *HSP70* (Lo et al., 2007) and *HSC70* (Chan et al., 2014) transcript levels are high at early vitellogenic stage but decrease during ovarian maturation. The *M. ensis* HSC70 was further demonstrated as a negative regulator for *Vg* gene expression (Chan et al., 2014). In our study, however, ovarian *Lv-HSP40* mRNA was observed to increase continuously during gonadal development, and hepatopancreatic *Lv-HSP40* mRNA also exhibited a slight raise between stage II and stage III (Figure 6A). In contrast, the hepatopancreatic/ovarian mRNA expressions of *Lv-HSP70* (Figure 6B), the cofactor of HSP40, were stable throughout the process of ovarian development. These results indicate that the expression of *Lv-HSP40* during ovarian maturation is independent of *Lv-HSP70*. The RNAi approach was applied to investigate the potential role of Lv-HSP40 in ovarian development in *L. vannamei*. In this case, siRNA HSP40-474 injection significantly delayed ovarian maturation and spawning (Figure 7C), and reduced the GSI (Figure 7D), indicating that Lv-HSP40 may play an important role in this process. However, ovarian development was not totally abolished by the silencing of *HSP40*, showing that the role of Lv-HSP40 is not irreplaceable. The participation of Lv-HSP40 in the ovarian maturation of *L. vannamei*. is independent of HSP70, based on the fact that the expression of this co-chaperone was not altered after RNAi of *Lv-HSP40* (Figure 7E). Furthermore, deposition of yolk protein, the hydrolysis product of *Vg*, is a prerequisite of vitellogenesis in *L. vannamei* (Chen et al., 2014; Chen H.Y. et al.,2018; Luo et al., 2015). However, neither the hepatopancreatic or ovarian *Vg* (Figure 7F), nor the ovarian *VgR* (Figure 7G) transcript levels changed after RNAi of *Lv-HSP40* for 24 h. It is speculated that Lv-HSP40 directly acting on the ovary and is irrelevant to the exogenous vitellogenesis occurred in hepatopancreas (Chen T. et al., 2018). Within the ovary, *Lv-HSP40* mRNA were majorly expressed in the follicle cells (Figure 4B), which provide nutrients and energy to the developing oocytes for growth and maturation. Although Lv-HSP40 performs its roles on the somatic cells but not germ cells in the ovary, it may affect the whole process of ovarian maturation (Figures 7C,D). Based on the fact that no morphological abnormalities were noticed in the ovarian cells after silence of *Lv-HSP40*, the precise mechanism for HSP40 regulation of ovarian development is needed further investigated.

In summary, a novel HSP40 has been identified in the important commercial cultured shrimp *L. vannamei* in the present study. The transcript expression profiles of *Lv-HSP40* have been characterized in different tissues, different embryonic and larval stages, different ovarian developmental stages, and under extreme conditions of temperature, pH and salinity. Based on the RNAi approach, this study is the first to provide evidence for the significant roles of Lv-HSP40 in ovarian maturation in *L. vannamei*. As a whole, the results from this study are important in opening a new research area for HSP40 as a molecular chaperone in controlling multiple physiological processes in crustaceans, which is a basic molecular function that benefits the improvement of shrimp aquaculture.

## Author Contributions

TC, CH, PL, and CR conceived and designed the experiments. TC, TLi, HL, TLu, JL, WH, XJ, and JZ performed the experiments. TC, TLi, HS, AY, and CR analyzed the data. AY, CH, PL, and CR contributed to reagents, materials, and analysis tools. TC, TLi, PL, and CR wrote the paper.

## Conflict of Interest Statement

The authors declare that the research was conducted in the absence of any commercial or financial relationships that could be construed as a potential conflict of interest.
