# Supplementary material for: Heat Shock Protein 40 (HSP40) in Pacific White Shrimp (Litopenaeus vannamei): Molecular Cloning, Tissue Distribution and Ontogeny, Response to Temperature, Acidity/Alkalinity and Salinity Stresses, and Potential Role in Ovarian Development
Source: Front Physiol. 2018 Dec 12;9:1784. doi: 10.3389/fphys.2018.01784 (PMC6299037; doi:10.3389/fphys.2018.01784)
Supplement: Supplementary file 1 [file Table_1.DOCX]

**Supplementary data. 1** (Suppl. 1) A unigene of 1,075 bp in a *L. vannamei* Illumina transcriptome that shares high sequence homology with *HSP40* in other species.

GAAGCCCACAGCAACAACTGAAGAACTTAAGAAGGCGTACAGAAAACTTGCACTGAAGTATCATCCTGATAAGAATCCAAATGAAGGAGAAAAGTTCAAACTGATTTCACAAGCTTATGAGGTACTGAGCAATGAGGAAAAACGTGCAATTTATGACCAAGGTGGAGAACAGGCCCTCAAGGAAGGCGGCACTGGAGGTGGTGGCTTCACATCACCAATGGATATCTTTGAGATGTTCTTTGGGGGTGGTTCAAGACGAAGCAGAGAAAAGAAGGTTAAGGATGTCATCCATCAAATGAGTGTTTCACTGGAGGAGTTGTACAATGGTGCAGTCAGAAAGCTAGCACTGCAGAAGCACGTTATCTGCAGCAAGTGTGAGGGGCAGGGTGGTAAGAAGCCCCCAGAGAAGTGCCCTTCTTGCCGCGGAACAGGAATGCAGGTCCGGATTCAACAATTAGGCCCCGGCATGGTGTCACAAGTGCAGAGTATGTGTGGCGAGTGCCGTGGTCAGGGAGAACGCATTAATCCTAAAGACAGATGCAAAACCTGTGAAGGCCGGAAAGTTGTTAAAGACCGCAAAATCCTAGAGGTTCATGTAGACAAAGGCATGGAAGATGGTCAGAAAGTTGTATTTTCTGGCGAGGGTGATCAGGAACCTGGCTTAGAACCTGGTGACATCATCATCGTTCTGGATGAGAAGGAGCATGCAACTTTCAAACGTGTGAACAGTGATCTCACAATGCAGATGCACATTTCTCTTGTAGAAGCATTATGTGGATTCCAGAAGCCTATCAAGACCTTAGATGACAGAACCATTGTTATTAGTGCAATCCCAGGTGAAGTAATAAAGAATGCTGAAGTGAAGAGTGTCCTTGGTGAGGGCATGCCTCAATACAAGAATCCATTCGAAAAAGGACGTTTACTCATTCAATTCTTGGTGGACTTCCCACCTCATATTTCATCAGATCGTATTGCTAAGTTGGAAAAAATATTGCCTGCAAGGCCTGAAGTAATGATTCCAGATGATTGTGAAGAATGCGAGCTAGCTGAAATTGATAGGTCACAACGAAGCCGA
