# Supplementary material for: Heat Shock Protein 40 (HSP40) in Pacific White Shrimp (Litopenaeus vannamei): Molecular Cloning, Tissue Distribution and Ontogeny, Response to Temperature, Acidity/Alkalinity and Salinity Stresses, and Potential Role in Ovarian Development
Source: Front Physiol. 2018 Dec 12;9:1784. doi: 10.3389/fphys.2018.01784 (PMC6299037; doi:10.3389/fphys.2018.01784)
Supplement: Supplementary file 2 [file Table_2.DOCX]

**Supplementary data. 2** (Suppl. 2) The sequence of a 585 bp DIG-labeled DNA probe against *Lv-HSP40* mRNA for *in situ* hybridization.

GGGCAGGGTGGTAAGAAGCCCCCAGAGAAGTGCCCTTCTTGCCGCGGAACAGGAATGCAGGTCCGGATTCAACAATTAGGCCCCGGCATGGTGTCACAAGTGCAGAGTATGTGTGGCGAGTGCCGTGGTCAGGGAGAACGCATTAATCCTAAAGACAGATGCAAAACCTGTGAAGGCCGGAAAGTTGTTAAAGACCGCAAAATCCTAGAGGTTCATGTAGACAAAGGCATGGAAGATGGTCAGAAAGTTGTATTTTCTGGCGAGGGTGATCAGGAACCTGGCTTAGAACCTGGTGACATCATCATCGTTCTGGATGAGAAGGAGCATGCAACTTTCAAACGTGTGAACAGTGATCTCACAATGCAGATGCACATTTCTCTTGTAGAAGCATTATGTGGATTCCAGAAGCCTATCAAGACCTTAGATGACAGAACCATTGTTATTAGTGCAATCCCAGGTGAAGTAATAAAGAATGCTGAAGTGAAGAGTGTCCTTGGTGAGGGCATGCCTCAATACAAGAATCCATTCGAAAAAGGACGTTTACTCATTCAATTCTTGGTGGACTTCCCACCTCATATTTCATCA
