# Supplementary material for: Heat Shock Protein 40 (HSP40) in Pacific White Shrimp (Litopenaeus vannamei): Molecular Cloning, Tissue Distribution and Ontogeny, Response to Temperature, Acidity/Alkalinity and Salinity Stresses, and Potential Role in Ovarian Development
Source: Front Physiol. 2018 Dec 12;9:1784. doi: 10.3389/fphys.2018.01784 (PMC6299037; doi:10.3389/fphys.2018.01784)
Supplement: Supplementary file 3 [file Table_3.DOCX]

**Supplementary data. 3** (Suppl. 3) The siRNA sequences used in this study.

| Name |  | Sequences |
| --- | --- | --- |
| ***HSP40-42*** | sense  antisense | GCCCACAGCAACAACUGAATT  UUCAGUUGUUGCUGUGGGCTT |
| ***HSP40-474*** | sense  antisense | GCAGGUCCGGAUUCAACAATT  UUGUUGAAUCCGGACCUGCTT |
| ***HSP40-825*** | sense  antisense | GCCUAUCAAGACCUUAGAUTT  AUCUAAGGUCUUGAUAGGCTT |
| ***NC-siRNA*** | sense  antisense | UUCUCCGAACGUGUCACGUTT  ACGUGACACGUUCGGAGAATT |
