# Supplementary material for: Heat Shock Protein 40 (HSP40) in Pacific White Shrimp (Litopenaeus vannamei): Molecular Cloning, Tissue Distribution and Ontogeny, Response to Temperature, Acidity/Alkalinity and Salinity Stresses, and Potential Role in Ovarian Development
Source: Front Physiol. 2018 Dec 12;9:1784. doi: 10.3389/fphys.2018.01784 (PMC6299037; doi:10.3389/fphys.2018.01784)
Supplement: Supplementary file 4 [file Table_4.DOCX]

**Supplementary data. 4** (Suppl. 4) Real-time PCR conditions and primer sequences used in this study.

| Gene Target/Accession No.  (Primer Sequences, 5’-3’) | PCR Condition | | | | | | Product Size |
| --- | --- | --- | --- | --- | --- | --- | --- |
|  | Denaturing | Annealing | Extension | Detection | Cycle no. | Tm |  |
| ***HSP40*/ MH932106** | 95°C | 60°C | 72°C | 84°C | 40 | 88°C | 198 bp |
| TTCTGGATGAGAAGGAGCAT | 5 s | 30 s | 30 s | 20 s |  |  |  |
| TGCCCTCACCAAGGACACTCT |  |  |  |  |  |  |  |
| ***HSP70*/ AY645906** | 95°C | 55°C | 72°C | 84°C | 40 | 89°C | 264 bp |
| TGCGAAGCGTACCCTGTC | 5 s | 30 s | 30 s | 20 s |  |  |  |
| TTGCCGTTGAAGAAGTCCTG |  |  |  |  |  |  |  |
| ***Vg*/ KC962398** | 95°C | 62°C | 72°C | 79°C |  |  |  |
| GGTGTTGCTGTTGCTGCTGTGAA | 5 s | 30 s | 30 s | 20 s | 40 | 84°C | 239 bp |
| TTGACTAACTGAGATGAAGAGAAC |  |  |  |  |  |  |  |
| ***VgR*/ Suppl.5** | 95°C | 58°C | 72°C | 79°C |  |  |  |
| GCTCTGACGAAAGCCCTTATGC | 5 s | 30 s | 30 s | 20 s | 40 | 84°C | 213 bp |
| CACCGTTTCCCAAGCAGGTTT |  |  |  |  |  |  |  |
| ***β-actin*/ JF288784** | 95°C | 60°C | 72°C | 86°C |  |  |  |
| CCGGCCGCGACCTCACAGACT | 5 s | 30 s | 30 s | 20 s | 35 | 91°C | 236 bp |
| CCTCGGGGCAGCGGAACCTC |  |  |  |  |  |  |  |
